# Coinfection with cryptococcus and aspergillus in an immunocompetent adult: A case report

**DOI:** 10.1097/MD.0000000000012612

**Published:** 2018-09-28

**Authors:** Qi Wang, Zhaoyong Wang, Yuqiu Hao, Wei Li, Tong Xin, Mo Chen, Peng Gao

**Affiliations:** a Department of Respiratory and Critical Care Medicine; b Department of Pathology, the Second Hospital of Jilin University, Changchun, Jilin, China.

**Keywords:** aspergillosis, *aspergillus*, cryptococcosis, *Cryptococcus neoformans*, healthy host

## Abstract

**Rationale:**

*Aspergillus* and *Cryptococcus* exposure can cause serious secondary infections in human lungs, especially in immunocompromised patients or in conjunction with a chronic disease caused by low disease resistance. Primary invasive fungal infections are clinically rare; therefore, coexistence of 2 fungi at an infection site is uncommon. This paper reports a case of healthy male who was diagnosed with both *Cryptococcus neoformans* and *Aspergillus* infections.

**Patient concerns:**

A healthy 33-year-old male office worker was admitted to the Second Hospital of Jilin University for hemoptysis. A chest computed tomography (CT) scan showed a cavity, which was formed by the thick dorsal wall of the lower left lobe with an irregular inner wall and burr changes around the lesion.

**Intervention:**

After 1.0 week of antibiotic and antituberculosis treatment, the hemoptysis symptoms remained. A resection of the left lower lobe was performed.

**Diagnoses:**

The postoperative pathological reports indicated the presence of both *Aspergillus* and *Cryptococcus*. The 2 fungal lesions were separate but within the same location.

**Outcomes:**

After treatment, the patient no longer had hemoptysis.

**Lessons:**

The current study indicated that fungi can infect not only immunocompromised patients but also healthy people, and that there can be 2 separate fungal infections at the same infection site.

## 1 Introduction

Over the past decade, more than 200 fungi have been identified as human pathogens, of which *Candida*, *Aspergillus*, and *Cryptococcus* are the most opportunistic fungi that cause infections worldwide,^[1]^ especially in immunocompromised or immunosuppressed patients. Invasive pulmonary aspergillosis (IPA) is the most serious type of pulmonary *Aspergillus* infection, and becomes a secondary infection to pulmonary tuberculosis (TB), bronchiectasis, lung abscess, pulmonary cysts, and cancer chemotherapy. Even though the host's immune function is low in these patients, primary fungi infection is rare.^[2]^ Pulmonary cryptococcosis (PC) is also more common in patients with immunodeficiency. Early studies suggested that this infection was a common complication in patients with immunodeficiency; however, a recent study has shown that the incidence in healthy people without immunodeficiency is on the rise.^[3]^ PC without a primary lung disease or abnormal lung structure is known as “primary” PC. Primary PC is more common in community-acquired infections even though the host might have no risk factors for fungal infection, and cases in which a healthy host is infected with *Aspergillus* or *Cryptococcus neoformans* are rare. Pulmonary invasive *Aspergillus* infection together with *C neoformans* infection is even more uncommon. Here, we report a case of a void-type (abscess-like) *Aspergillus* infection that was not associated with *Cryptococcus* infection and in which the 2 pathogens were present in adjacent but separate lesions. Although the patient was not immunocompromised, he was diagnosed with cryptococcosis with aspergillosis by lung biopsy using video-assisted thoracoscopic surgery (VATS) and was successfully treated as a result of early intervention.

## 2 Case presentation

**Figure 1:**
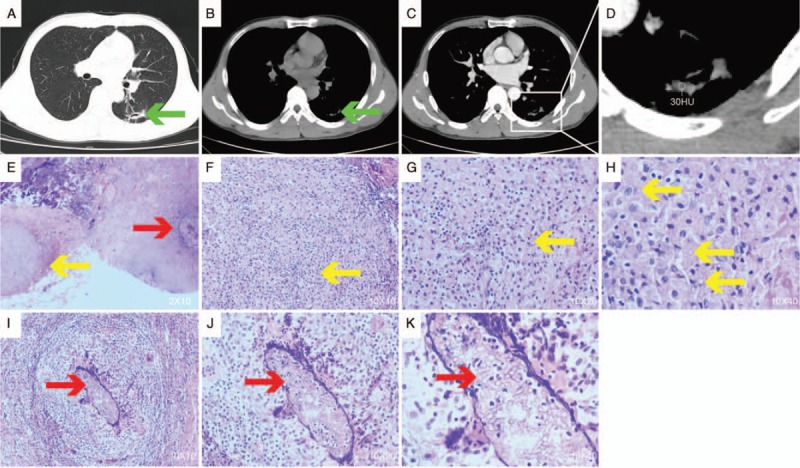
Chest radiological changes and pathological findings. (A) 3.0-cm increase in density with clear boundaries in the left upper lobe; (B, C) enhanced and non-enhanced computed tomography (CT) images of 2 nodes of the pulmonary mediastinal window; (D) enlarged view of the pulmonary mediastinum window nodules showing 2 nodules with irregular and well-defined borders in the upper left lobe. The larger nodule has an irregular border with a uniform density of 2.4 cm, in which cavities are visible. The smaller 0.7-cm nodule is on the left; (E) surgically resected lung biopsy. Pathology suggested that both fungal foci existed separately (hematoxylin and eosin stain, original magnification 20×). The yellow arrow tips are cryptococcal lesions, and the red arrow tips are aspergillosis lesions; (F–H) visible cryptococcosis; (I–K) *Aspergillus* can be seen, and there is a large number of chronic inflammatory cells and histiocytic infiltration, lymphoid follicle formation, and a large amount of local necrosis (hematoxylin and eosin stain, original magnification 100×, 200×, 400×).

A healthy 33-year-old male office worker with no history of TB, surgery, or medication was hospitalized in the Second Hospital of Jilin University on October 27, 2016, for intermittent bloody sputum for 3.0 months that was left untreated. The condition became aggravated 1.0 week before admission. The hemoptysis showed bright-red or dark-red blood of 3.0 to 5.0 mL/day. The patient did not have a significant fever, night sweats, or weakness. Wet rales were heard during auscultation of the lower left lung; however, the results of other physical examinations were normal. A chest computed tomography (CT) scan showed mild inflammation in the lower left lobe. The thick dorsal wall of the lower left lobe formed a cavity with an irregular inner wall, a thick dorsal wall, and burr changes around the lesion, and the upper lobe showed evidence of pulmonary emphysema. Laboratory tests were negative for bacterial growth in the sputum culture, sputum acid-fast bacilli, T-SPOT, (1→3)-β-D-glucan test, and galacto-mannan test; they also showed a normal erythrocyte sedimentation rate. Initially, treatment with empirical antibiotics combined with experimental anti-TB drugs showed no significant improvement in symptoms. After a bronchoscopic lung biopsy and exfoliative cytology, the patient's exfoliative cells showed pathological changes in the epithelial cells, phagocytes, and nonmalignant cells. After 1.0 week of antibiotic treatment, the hemoptysis symptoms remained. An enhanced thoracic CT was then performed on the patient and cavitary nodules containing a fungus ball, which was not enhanced (Fig. 1A–D). A video-assisted thoracoscopic surgery (VATS) left lower-lobe resection was performed under general anesthesia. The postoperative pathological reports indicated a large number of chronic inflammatory cells and tissue infiltration, lymphoid follicle formation, a large amount of local necrosis, multinucleated cells, and fungi. The fungal morphology was mainly that of *Aspergillus* (Fig. 1E, I, J, K), surrounded by fibers, with granulomatous inflammation and spore-like structural necrosis. The size of the spores was consistent with that of *Cryptococcus* (Fig. 1E–H). The fungal infections coexisted but the fungal foci of each was separate (Fig. 1E). The result of the latex agglutination test for cryptococcal capsular polysaccharide antigen was positive. An acid-fast stain was also applied; however, TB was not found. The patient was eventually conclusively diagnosed as having invasive pulmonary aspergillosis with PC. He was treated with amphotericin B and fluconazole until being discharged 7.0 days after surgery, and was prescribed oral amphotericin B and fluconazole daily for 4.0 months after discharge. The patient continues his outpatient visits. Chest CT scan demonstrated no obvious abnormality except the postsurgical chordae shadow in the left lower lobe 1.0 month after discharge. The follow-up was conducted for 1.0 year.

## 3 Discussion

In recent years because of the extensive use of broad-spectrum antimicrobial drugs, glucocorticoids, and immunosuppressive agents, organ transplantation and extensive tumor oncology and chemotherapy, as well as the acquired immune deficiency syndrome epidemic, have resulted in increased risk factors for IPA.^[4]^ Previously reported secondary IPA is easily correlated with an immune-deficiency disease, and long-term use of glucocorticoids or immunosuppressive agents that lead to immune dysfunction. The occurrence of acute IPA in a previously healthy subject is rather uncommon.^[5]^ PC is an acute, subacute, or chronic pulmonary fungal disease caused by a cryptococcal infection. These opportunistic pathogens will infect those with impaired immunity. In addition to patients with the human immunodeficiency virus (HIV), those with a variety of underlying diseases, such as diabetes, cancer, rheumatoid immune diseases, lung disease, and renal failure hemodialysis, are also susceptible to primary PC. Around 20% to 30% of healthy individuals with normal immune function can also be infected. In summary, multiple fungal pathogens infect immunocompromised individuals and are uncommon in patients with no evidence of comorbidity or immunosuppression. *Aspergillus* and cryptococcal coinfection in the same lesion is also extremely rare. Through a PUBMED database search, we found only 5 reports that described the coexistence of these 2 fungi. Only 2 of these patients were in good health^[6,7]^; the other 3 were immunocompromised, comprising a 59-year-old male with HIV who also had pulmonary TB,^[8]^ a 33-year-old female suffering from systemic lupus erythematosus (SLE) and receiving immunosuppressive agents and sugar corticosteroid treatment,^[9]^ and a 74-year-old male with a history of old-fashioned hollow TB.^[7]^ Lung cavities were present in all of these patients. Previous reports suggested that bronchodilation and TB cause voids that usually harbor *Aspergillus* or *Cryptococcus*, the 2 fungi in the mixed infection in these patients.^[7]^ In the 5 cases, the formation of lung cavities might have been the basis and premise for the 2 fungal infections. In 4 of the 5 cases, central necrosis of cryptococcosis played a major role in the formation of empty lung cavities. Aspergillosis was secondary to the empty cavities. In one case, there was active TB, and another was coinfected with cryptococcosis and *Aspergillus* in an old TB cavity.

In our study, the 33-year-old worker was healthy without a history of TB, obstructive pulmonary voids, and bronchiectasis, and without clinical data on immunosuppression. In this case, hemoptysis led to hospitalization; however, the clinical manifestation lacked specificity. Blood and sputum tests after admission did not find any fungal infections. The results of CT scans of the lungs showed no improvement; therefore, anti-infection and anti-TB treatment was not prescribed. After performing VATS, the patient was indeed confirmed to be coinfected with *C neoformans* and *Aspergillus*. Unexpectedly, the pathology results showed that the invasive *Aspergillus* infection in the patient resulted in the formation of cavities, although *Cryptococcus* did not exist in the cavities; therefore, the 2 fungal infections were adjacent but separate. Finally, the infections were successfully cured by thoracotomy and antifungal treatment. The review of this patient is important in clinical settings because it shows that fungi can infect healthy people as well as immunocompromised patients, and that there can be 2 types of fungal infections that coexist.

## Author contributions

QW, ZW, and YH carried out the data collection, literature review and drafting of the manuscript. WL contributed to the drafting of the manuscript and aided in the literature review. TX and MC participated in the data collection and the drafting of the manuscript. PG helps to draft the manuscript and revised the final version of the manuscript. All authors read and approved the final manuscript.

**Data curation:** Tong Xi.

**Formal analysis:** Yu qiu Hao, Mo Chen.

**Investigation:** Wei Li.

**Software:** Zhao yong Wang.

**Writing – original draft:** Qi Wang.

**Writing – review & editing:** Peng Gao.

Peng Gao orcid: 0000-0001-7328-1235
